# Circulating microRNAs as Specific Biomarkers for Breast Cancer Detection

**DOI:** 10.1371/journal.pone.0053141

**Published:** 2013-01-03

**Authors:** Enders K. O. Ng, Rufina Li, Vivian Y. Shin, Hong Chuan Jin, Candy P. H. Leung, Edmond S. K. Ma, Roberta Pang, Daniel Chua, Kent-Man Chu, W. L. Law, Simon Y. K. Law, Ronnie T. P. Poon, Ava Kwong

**Affiliations:** 1 Department of Surgery, The University of Hong Kong, Hong Kong, China; 2 Department of Clinical Oncology, The University of Hong Kong, Hong Kong, China; 3 Department of Molecular Pathology, Hong Kong Sanatorium and Hospital, Hong Kong, China; 4 Biomedical Research Center, Sir Runrun Shaw Hospital, Medical School of Zhejiang University, Hangzhou, China; The University of Kansas Medical Center, United States of America

## Abstract

**Background:**

We previously showed microRNAs (miRNAs) in plasma are potential biomarkers for colorectal cancer detection. Here, we aimed to develop specific blood-based miRNA assay for breast cancer detection.

**Methodology/Principal Findings:**

TaqMan-based miRNA profiling was performed in tumor, adjacent non-tumor, corresponding plasma from breast cancer patients, and plasma from matched healthy controls. All putative markers identified were verified in a training set of breast cancer patients. Selected markers were validated in a case-control cohort of 170 breast cancer patients, 100 controls, and 95 other types of cancers and then blindly validated in an independent set of 70 breast cancer patients and 50 healthy controls. Profiling results showed 8 miRNAs were concordantly up-regulated and 1 miRNA was concordantly down-regulated in both plasma and tumor tissue of breast cancer patients. Of the 8 up-regulated miRNAs, only 3 were significantly elevated (p<0.0001) before surgery and reduced after surgery in the training set. Results from the validation cohort showed that a combination of miR-145 and miR-451 was the best biomarker (p<0.0001) in discriminating breast cancer from healthy controls and all other types of cancers. In the blind validation, these plasma markers yielded Receiver Operating Characteristic (ROC) curve area of 0.931. The positive predictive value was 88% and the negative predictive value was 92%. Altered levels of these miRNAs in plasma have been detected not only in advanced stages but also early stages of tumors. The positive predictive value for ductal carcinoma in situ (DCIS) cases was 96%.

**Conclusions:**

These results suggested that these circulating miRNAs could be a potential specific biomarker for breast cancer screening.

## Introduction

Breast cancer is one of the three most commonly diagnosed cancers among women, accounting for about 30% of patients [1]. Screening for breast cancer allows early stage diagnosis of the malignancy and hence has potential to reduce mortality. In the past decades, despite the dedication of research and resources to the development of biomarkers for diagnosis and prognosis, unpredictable response and development of resistance to adjuvant therapy remain as major challenges in breast cancer management. Although mammography diagnosis for breast cancer is the currently used noninvasive screening tool, the cost incurred and expertise required for mammography has hampered the wide application of this procedure. In addition, it has limited sensitivity in its use in young women and women with dense breasts. On the other hand, alternative methods such as ultrasound screening has very operator dependent sensitivity and tumor markers such as CA15.3 and carcinoembryonic antigen (CEA) are also non-specific and has limited sensitivity and specificity [2]. Thus, there is still a pressing need to develop a cost-effective and accurate screening method for this cancer.

Recently, the emergence of small non-protein-coding RNAs, microRNAs (miRNAs), playing important roles in oncogenesis has opened new opportunities for early cancer diagnosis [3], [4]. MiRNAs are 19–25-nucleotides regulatory non-coding RNA molecules that regulate the expressions of a wide variety of genes by sequence-specific base pairing on the 3′ untranslated regions of the target mRNA resulting in mRNA degradation or inhibition of translation. Evidence suggests that miRNA expression profiles can cluster similar tumor types together more accurately than the expression profiles of protein-coding mRNA genes [5]. Furthermore, miRNA expression signatures have been used to predict prognosis [6], [7]. Importantly, expression of some miRNAs correlated with the molecular subtypes and with two major features of breast cancer (grade and ER status) [8]. As a screening tool acceptable for general population, it would be desirable to detect cancer accurately without resorting to an invasive procedure. Recently, several reports have suggested that circulating miRNAs are stable and detectable in serum/plasma and the levels of some miRNAs specifically elevated in the patients with tongue [9], lung cancer [10], prostate cancer [11], ovarian cancer [12], breast cancer [13], [14] and colorectal cancer [15], [16]. These findings suggest that blood-based miRNAs could emerge as revolutionary sources of biomarker for breast cancer diagnosis. Previously, we have shown that miRNAs in plasma are promising biomarkers for colorectal cancer detection [15]. Similarly, we here used a systemic strategy to identify miRNAs in plasma for breast cancer detection and utilize such miRNA biomarkers for the development of a sensitive and specific blood-based diagnostic assay.

## Materials and Methods

### Ethics Statement

The study was performed in accordance with the Declaration of Helsinki. Written informed consent was obtained from all participants involved in this study. This study was approved by the Institutional Review Board of the University of Hong Kong/Hospital Authority West Cluster and other contributing hospitals, Hong Kong.

### Study Design and Patients

**Figure 1:**
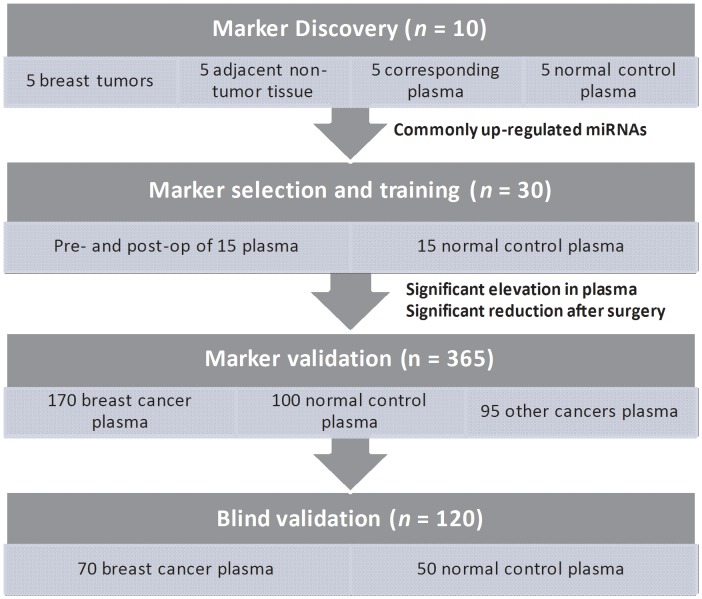
Schematic diagram of the workflow of this study.

A total of 260 patients who have been clinically diagnosed as breast cancer at the time of participation were recruited between 2008 and 2010 from Queen Mary Hospital and Tung Wah Hospital. A total of 170 normal control subjects were recruited from Queen Mary Hospital and Tung Wah Hospital and were pathologically confirmed not to have breast cancer and no history of other cancers. This study is divided into three stages (Figure 1): Marker discovery, Marker selection and training, Marker validation and Blind validation. *Marker discovery*: Preoperative plasma, corresponding breast tumor and their adjacent non-tumor breast tissues from 5 female untreated breast cancer patients were collected. Five age-matched pathologically confirmed non-breast cancer female normal control subjects were used as control. MiRNA profiling was performed on (a) breast cancer plasma, (b) normal plasma, (c) tumor, and (d) adjacent non-tumor tissue of those patients. By comparing miRNA profiles from the 4 groups, commonly up-regulated and down-regulated miRNAs in both plasma and tumor were identified. *Marker selection and training*: Plasma from 15 female breast cancer patients before and after 14 days after surgery were collected together with 15 pathologically confirmed non-breast cancer control subjects. Putative miRNA markers identified in stage I were validated on these samples. For those up-regulated markers proceeding to marker validation, the preoperative plasma levels of the marker must be significantly elevated and be significantly reduced after tumor resection. *Marker validation*: Plasma was collected from a case-control group (Queen Marry Hospital) of 170 untreated female breast cancer patients. Plasma from 60 normal female control subjects and 40normal male control subjects were also collected. Putative markers are verified on these set of samples. Plasma from colorectal cancer (CRC), esophagus cancer (EC), gastric cancer (GC), hepatocellular carcinoma (HCC) and lung cancer (LC) patients were also included to examine the specificity of the makers. *Blind validation*: Plasma was collected from an independent group (Tung Wah Hospital) of 70 untreated female breast cancer patients and 50 normal female subjects were collected. Tumors were staged according to the tumor-node-metastasis (TNM) staging system of WHO. Written informed consent was obtained from all participants involved in this study. This study was approved by the Institutional Review Board of The University of Hong Kong/Hospital Authority West Cluster, Hong Kong.

### Samples Processing and MicroRNA Extraction

Total RNA was extracted from tissues using the TRIzol® reagent (Invitrogen) following the manufacturer’s instructions. Blood samples were centrifuged at 1600 × g for 10 minutes at 4°C, and plasma was carefully transferred into new tubes followed by further centrifugation at 16000 × g for 10 minutes at 4°C. Total RNA containing small RNA was extracted from 500 µl of plasma using Trizol LS reagent (Invitrogen) and miRNeasy Mini Kit (Qiagen, Hilden, Germany) according to the manufacturer's protocol with the following modifications [15]: 1 ml Trizol LS reagent was added to 500 µl plasma samples. After phase separation, 1.5 volume of 100% ethanol was added to the aqueous phase and the mixture was loaded into miRNeasy column (Qiagen, Hilden, Germany) according to the manufacturer's instructions. DNase treatment was carried out to remove any contaminating DNA (RNase-Free DNase Set, Qiagen, Hilden, Germany). The final elution volume was 30 µl. The concentration of all RNA samples were quantified by NanoDrop 1000 (Nanodrop).

### Plasma miRNA Profiling

Profiling was performed using TaqMan Array Human MicroRNA Panels A and B (Applied Biosystems, Carlsbad, CA, USA) including the respective reverse-transcription primers, PCR primers, and TaqMan probe. For each assay, 150 ng of total RNA extracted from plasma or tissue samples was used for the reverse-transcription reaction with a pool of Megaplex RT primers according to the manufacturer's protocol. TaqMan-based qPCR profiling was performed using TaqMan Universal PCR Master Mix (Applied Biosystems) in ABI PRISM 7900 HT system (Applied Biosystems) according to the manufacturer’s instructions.

### Plasma miRNA Quantitative PCR

SYBR green qRT-PCR assay was used as previously described [15]. In brief, 50 ng of plasma RNA was polyadenylated and reverse transcribed to cDNA using miScript Reverse Transcription kit (Qiagen). Real-time qPCR was performed using miScript SYBR Green PCR kit (Qiagen) in ABI PRISM 7900 HT system. The miRNA-specific forward primers sequences were designed based on the miRNA sequences obtained from the miRBase database. The miRNA-specific primer sequences for qPCR were listed in Table S1. Each sample was run in duplicates for analysis. The expression levels of miRNAs were normalized to *RNU6B* (U6 small nuclear RNA) as described previously [15]. Fold change of miRNA was calculated by the equation 2^–ΔΔCt^. ΔCt was calculated by subtracting the Ct values of *RNU6B* from the Ct values of the miRNA of interest. ΔΔCt was then calculated by subtracting ΔCt of the control from ΔCt of cancer.

### Statistical Analysis

The significance of plasma miRNA levels was determined by Mann-Whitney test, Wilcoxon test, χ^2^ test or Kruskal-Wallis test where appropriate. The sensitivity and specificity were calculated according to the standard formulas. Multivariate logistic regression model was established and leave-one-out cross validation to find the best logistic model. ROC curves were established for discriminating patients with or without breast cancer. The optimal sensitivity and specificity from ROC curves were determined by a commonly used method [17]. All *P-*values are two-sided and less than 0.05 was considered statistically significant. All statistical calculations were performed by GraphPad PRISM 5 software (GraphPad Software, La Jolla, CA).

## Results

### Patient Characteristics

From December 2008 through September 2010, a total of 525 participants including 260 breast cancer patients, 170 normal control subjects, 20 CRC, 20 GC, 20 EC, 15 LC, and 20 HCC patients were recruited. Patient characteristics were summarized in Table 1. There were no significant differences of age between breast cancer patients and controls. The sex distribution in control group was 40∶130 (male: female).

**Table 1:** Patient characteristics.

	BC(n = 185)	Healthy(n = 145)	CRC(n = 20)	EC(n = 20)	GC(n = 20)	LC(n = 15)	HCC(n = 20)
Characteristic	No. ofpatients	No. ofpatients	No. ofpatients	No. ofpatients	No. ofpatient	No. ofpatient	No. ofpatient
Age (years)							
Mean	59	58	65	66	61	65	64
Median (range)	60 (36–84)	61 (40–85)	62 (54–80)	72 (50–86)	63 (45–78)	62 (55–78)	65 (56–78)
Gender							
Male	–	40	10	8	8	8	9
Female	185	105	10	12	12	7	11
TNM stage (Discovery set, n = 5)							
I	3	–	–	–	–	–	–
III	2	–	–	–	–	–	–
TNM stage (Training set, n = 15)							
I	1	–	–	–	–	–	–
II	7	–	–	–	–	–	–
III	2	–	–	–	–	–	–
TNM stage (Validation set, n = 120)							
DCIS/TIS	27	–	–	–	–	–	–
I	31	–	–	–	–	–	–
II	34	–	–	–	–	–	–
III	23	–	–	–	–	–	–
IV	5	–	–	–	–	–	–
TNM stage (Blind validation set, n = 45)							
I	15	–	–	–	–	–	–
II	20	–	–	–	–	–	–
III	10	–	–	–	–	–	–

BC: breast cancer; CRC: colorectal cancer; EC: esophagus cancer; GC: gastric cancer; LC: lung cancer; HCC: hepatocellular carcinoma.

### Plasma miRNA Marker Discovery

Previously, we have developed an approach for rapid and effective identification of tumor-derived miRNAs in plasma [15]. In this approach, the differential expression patterns between plasma and tumor tissue samples are compared so as to rule out plasma miRNAs predominantly derived from sources other than breast tumor cells. Using 2-fold expression difference as a cutoff level, 8 miRNAs (miR-16, miR-21, miR-27a, miR-150, miR-191, miR-200c, miR-210, miR-451) up-regulated and 1 miRNA (miR-145) down-regulated in both plasma and tumor tissues in breast cancer patients were identified (Table S2 and S3).

### Marker Selection and Training

**Figure 2:**
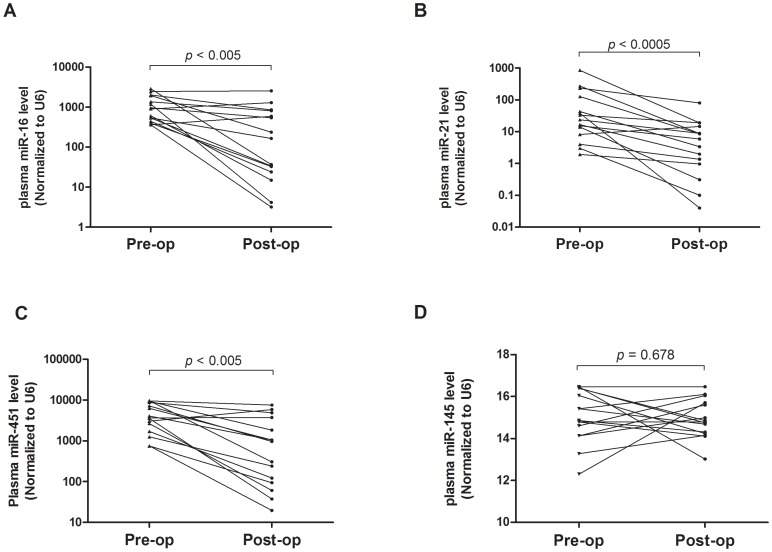
MicroRNA selection and validation by quantitative RT-PCR analysis. Changes of plasma levels of (*A*) miR-16, (*B*) miR-21, (*C*) miR-451 and (*D*) miR-145 in breast cancer patients (n = 15) before (pre-Op) and 14 days after (14 day post-Op) surgical removal of the tumor. Expression levels of the miRNAs (Log10 scale at Y-axis) are normalized to *RNU6B*. Statistically significant differences were determined using Wilcoxon tests.

To validate the putative markers identified from the profiling results, plasma levels of these miRNAs were measured by qRT-PCR assays. As described previously [15], *RNU6B* was used as an internal normalization control in plasma. To prove *RNU6B* is a reliable normalization control, plasma *RNU6B* level was evaluated in breast cancer patients. Our data demonstrated that no significant difference was observed in term of Ct values of *RNU6B* (p = 0.75) between control (n = 100) and breast cancer samples (n = 120) (Figure S1) suggesting that *RNU6B* constitutively expressed in plasma regardless of different disease conditions. Plasma levels of the 8 up-regulated miRNAs were validated by qRT-PCR on the 30 plasma samples (15 breast cancer patients and 15 controls). Our data indicated that all 9 miRNAs have 100% detection rate in plasma but only miR-16, miR-21 and miR-451 in breast cancer plasma were significantly elevated and plasma miR-145 level significantly lower when compared to controls (all *p-values*<0.001). To further verify whether miR-16, miR-21 and miR-451 are breast cancer-associated, their plasma levels were quantified on the postoperative plasma of the 15 breast cancer patients after 14 days surgical removal of the tumors. Our result showed that plasma levels of putative miR-16, miR-21 and miR-451 markers were significantly reduced in postoperative samples when compared to the preoperative samples (*p*<0.005; Figure 2) whereas no significant difference of miR-145 was obtained between pre- and post-operative plasma samples.

### Marker Validation

**Figure 3:**
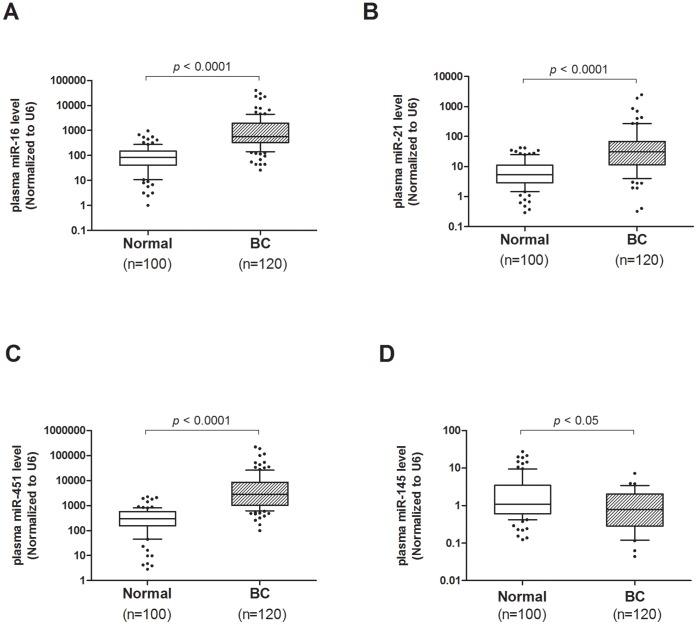
Large-scale validation of miR-16, miR-21, miR-451 and miR-145 on an independent group of plasma samples (n = 270). Box plot of plasma levels of (*A*) miR-16 and (*B*) miR-21 and (*C*) miR-451 (*D*) miR-145 in healthy normal (N) subjects (n = 100) and breast cancer patients (n = 170). Expression levels of the miRNAs are normalized to *RNU6B*. The lines inside the boxes denote the medians. The boxes mark the interval between the 25^th^ and 75^th^ percentiles. The whiskers denote the interval between the 10^th^ and 90^th^ percentiles. Filled circles indicate data points outside the 10^th^ and 90^th^ percentiles. Statistically significant differences were determined using Mann-Whitney tests.

**Figure 4:**
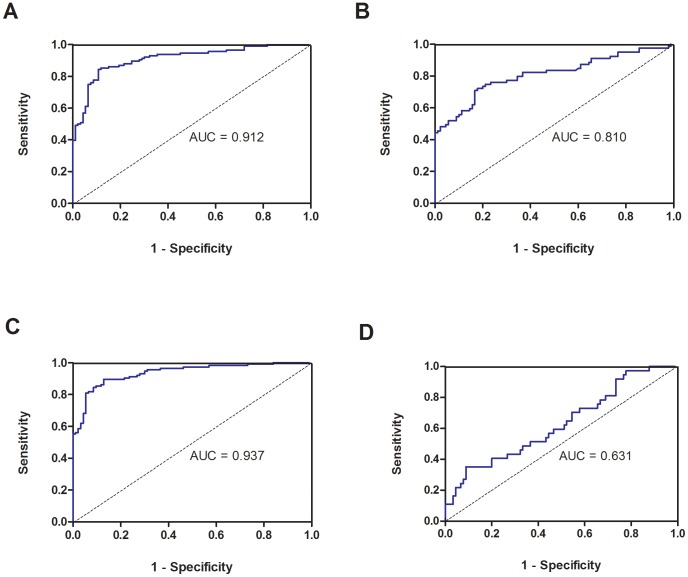
ROC curve analysis using (*A*) plasma miR-16, (*B*) plasma miR-21, (*C*) plasma miR-451 (*D*) miR-145 for discriminating breast cancer from normal subjects.

**Figure 5:**
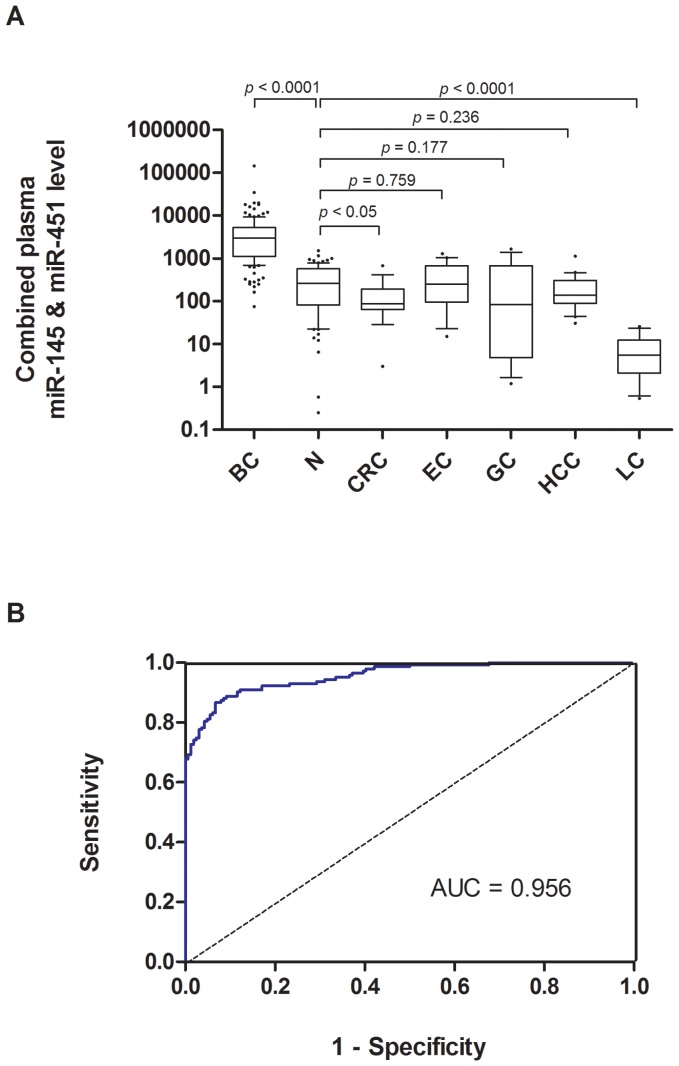
Large-scale validation of combined plasma miR-145 and miR451. (*A*) Box plot of combined plasma miR-145 and miR-451 levels in patients of breast cancer (BC), healthy normal (N), colorectal cancer (CRC), esophagus cancer (EC), gastric cancer (GC), hepatocellular carcinoma (HCC) and lung cancer (LC). Expression levels of the miRNAs (Log10 scale at Y-axis) are calculated by the equation 2^-ΔCt^. ΔCt was calculated by subtracting the Ct values of miR-145 from the Ct values of the miR-451. The lines inside the boxes denote the medians. The boxes mark the interval between the 25^th^ and 75^th^ percentiles. The whiskers denote the interval between the 10^th^ and 90^th^ percentiles. Filled circles indicate data points outside the 10^th^ and 90^th^ percentiles. Statistically significant differences were determined using Mann-Whitney tests. (*B*) ROC analysis using combined plasma miR-451 and miR-145 for discriminating breast cancer from normal subjects and other cancers.

To further verify the discriminating power of the four miRNAs identified for breast cancer diagnosis, plasma levels of miR-16, miR-21, miR-145 and miR-451 were assessed on an independent group of 270 plasma samples including 170 breast cancer patients, 60 female and 40 male normal controls. To examine whether those markers are gender specific, plasma levels were compared between the female and male healthy controls. Our data indicated similar plasma levels of those markers between female and male (Figure S2), suggesting their expressions are non-gender specific. Our results showed that all three up-regulated miRNAs were significantly higher in breast cancer patients than that of controls (all p*-values*<0.0001; Figure 3A–C) while miR-145 plasma level was significantly lower than that of controls (p<0.05; Figure 3D). Receiver operating characteristic (ROC) curve analyses showed that the ROC curve areas for miR-16 and miR-451 alone were 0.912 (95% CI 0.873-0.951) and 0.938 (95% CI 0.906-0.969), respectively (Figure 4A and 4C) while ROC curve area for miR-21 and miR-145 alone were 0.809 (95% CI 0.742−0.877) and 0.631 (95% CI 0.523−0.738), respectively (Figure 4B and 4D). Although miR-16 and miR-451 alone generated satisfactory ROC values, we have tried different marker combinations so as to obtain the best combination for diagnosis. Using multivariate logistic regression analysis, the best combination (p<0.0001) for breast cancer detection was miR-451 and miR-145. For this best marker combination calculation, we came up with a simple equation (2^−ΔCt^) in which ΔCt is calculated by subtracting the Ct values of miR-145 from the Ct values of the miR-451. As a result, we demonstrated that such combined marker of miR-451 and miR-145 plasma levels in breast cancer patients were significantly discriminated not only normal control subjects but also all other types of cancers recruited in this study (Figure 5A). Furthermore, such combined markers yielded a ROC curve area of 0.956 (95% CI 0.936−0.978; Figure 5B). The optimal sensitivity and specificity were 90.2% (95% CI 84.1%−94.5%) was 89% (95% CI 82.5%−92.9%) in discriminating breast cancer including benign tumors from normal control plus all other types of cancers. Using such plasma marker, the positive predictive value was 90% (153/170) and the negative predictive value was 94.3% (184/195). The odds ratio for cases with combined miRNAs being associated with cancer was 44.2 (95% CI 19.3−101.1).

To examine whether the plasma levels of miR-145 and miR-451 may be associated with tumor stages, patients stratified by TNM staging were assessed. Of the 170 breast cancer patients, combined plasma miR-145 and miR-451 levels did not vary significantly across benign, DCIS and various stage of the patient’s tumor (p = 0.32; Figure S3). However, significant differences were obtained when individual tumor stage, DCIS and benign tumors were compared with the normal controls. Unfortunately, benign tumors cannot be distinguished from invasive breast cancer by this plasma miRNA levels. Intriguingly, of all DCIS cases (n = 27) from our breast cancer cohort, this combined plasma marker generated a ROC value of 0.974 (95% CI 0.930−1.02). The optimal sensitivity and specificity were 94.1% and 98.0%, respectively, in discriminating DCIS cases from controls (Figure S4A). When DCIS patients were stratified into high, intermediate and low grades, the combined plasma marker indeed elevated significantly across DCIS grading (p<0.0001; Figure S4B). Unfortunately no correlation between plasma miRNA levels and across DCIS grading was obtained.

### Blind Validation

We further verified our markers on a blind validation cohort. Plasma levels of miR-145 and miR-451 were assessed on an independent blind cohort from a different hospital of 70 female breast cancer patients and 50 healthy controls. Our blind validation results showed that the positive predictive values for breast cancer were 88%. The negative predictive value of this marker was 92%. This marker yielded ROC value of 0.931 (95% CI = 0.886 to 0.977), optimal sensitivity was 83% (95% CI = 72% to 91%) and optimal specificity was 93% (95% CI = 81% to 98%; Figure S5).

## Discussion

In this study, we have identified significant elevation of the miR-16, miR-21, and miR-451 and significant reduction of miR-145 in the plasma of breast cancer patients. Intriguingly, the combination of plasma miR-145 and miR-451 levels provided the best markers for breast cancer prediction and yielded a ROC curve area of 96%. The optimal sensitivity was 90% and optimal specificity was 92% in discriminating breast cancer from control subjects including all other types of cancers recruited in this study. The odds ratio for the cases with combined miR-145 and miR-451 level being associated with breast cancer was 44.2. Thus, this finding compares favorably with one of the best results using other circulating biomarkers for breast cancer diagnosis [18]. In the blind validation, the positive predictive value was 88% and the negative predictive value was 92%. Importantly, of the 27 DCIS cases from our breast cancer cohort, the combined plasma marker generated surprisingly good diagnostic power with optimal sensitivity of 94% and specificity of 98% in discriminating DCIS cases from controls.

Strikingly, we here the first time showed that the best combination of miR-451 and miR-145 plasma levels are very specific to breast cancer and did not elevate in all other cancer types included in this study. Besides, elevation of plasma miRNAs has been detected in not only advanced stages of breast cancer but also early stages (pre-invasive stages) of breast cancer such as DCIS suggested that this marker might be useful for early diagnosis. Surprisingly, we obtained 97% ROC value, 94% sensitivity and 98% specificity in discriminating DCIS cases from healthy controls. Thus, further verification is worthy to be conducted in the future to elucidate the underlying mechanism of down-regulated miR-145 and up-regulated miR-451 in breast cancer.

Several studies have demonstrated that miR-451 was involved in cancer. One report found that miR-451 was significantly over-expressed between non-relapsed and relapsed head and neck squamous cell carcinoma patients [19]. Transfection of the doxorubicin-resistant breast cancer cells with miR-451 resulted in the increased sensitivity of cells to the drug, indicating that correction of altered expression of miRNA may have significant implications for therapeutic strategies aiming to overcome cancer cell resistance [20]. These findings support the role of miR-451 as a regulator of cancer proliferation and open new perspectives for the development of effective therapies for chemoradioresistant cancers [21]. Our findings in this study suggest that miR-451 is a potential marker for breast cancer detection. A recent study showed that increases in plasma miR-451 levels are caused by haemolysis [22]. Quantification of plasma miR-451 level showed that the level in whole blood are dominated by the miRNA content of red blood cells and that only a fraction is derived from plasma or PBMCs (<1%). To exclude the discrepancy in our study, we quantified serum miR-451 and miR-145 levels from 30 breast cancer patients and 30 healthy subjects in our plasma study cohort. Our data indeed showed that the combined miR-145 and miR-451 levels in serum from patients were significantly higher than that of healthy subjects (Figure S6). This data indeed suggests that the increases in plasma miR-451 levels are not caused by haemolysis.

Emerging studies showed that miR-145 is a putative tumor-suppressive gene that is down-regulated in several types of tumors [23], [24] and inhibits cell growth by targeting *c-Myc* [25] and *IRS-1* [26]. Furthermore, miR-145 is reported to target the pluripotency factors *OCT4*, *SOX2*, and *KLF4* and plays a key regulator in human stem cells [27]. Recently, Sachdeva *et al* further characterized miR-145 in different cancer cell lines and found that miR-145 functions as a tumor suppressor in a cell type–specific manner. This report showed that miR-145 is a tumor suppressor affecting invasion and metastasis [28].

Although miR-16 is not the best combination of plasma markers in this study, differential expression of miR-16 has been reported in several cancers including leukemia, pituitary adenomas, prostate carcinoma, lung cancer and head and neck [19], [29]-[31]. Our results that aberrant expression of plasma miR-16 in breast cancer patients support the notion that miR-16 is not suitable for normalization. Thus, the use of miR-16 as an internal normalization control for miRNA quantitation [32] in clinical specimens such as whole blood is still questionable.

Compared with other studies of miRNAs in diagnosing breast cancer [18], [33], [34], our study is unique because we identified a combination of miRNA markers which are very specific to breast cancer only, not other cancers such as gastric, lung and HCC. Interestingly, these plasma levels were significantly decreased in lung cancer when compared with breast cancer and even normal control. This may be probably because miR451 was recently found to be one of the most down-regulated miRNAs in non-small cell lung carcinoma (NSCLC) tissues [35]. Thus, further study on plasma miR-451 levels in NSCLC might provide clues on potential biomarker for lung cancer diagnosis. Furthermore, these miRNAs identified in our study are also useful as a diagnostic marker for early stage of cancer, DCIS. Our finding that differential expression signatures in plasma with only two miRNAs could discriminate breast cancer from normal subjects raises the possibility of using such marker to develop a blood-based screening test for breast cancer in the future. Furthermore, circulating miRNA as a diagnostic marker has advantages over circulating mRNA. Unlike screening for large number of RNA expression, a modest number of miRNAs might be sufficient to differentiate cancers from normal. Unlike plasma RNAs, miRNAs in plasma remain largely intact and have been proven to be more stable for detection [11], [36].

Although our results are promising, several limitations in this study are addressed: (i) as the sample size is still small, further validations in large cohorts or in different ethnic groups are recommended; (ii) it is uncertain whether this plasma miRNA elevation is specific for certain subtypes of breast cancer, and whether such marker can be used to differentiate sporadic from familial types such as BRCA mutation carriers. Thus, additional studies are necessary to compare their plasma levels with different subtypes; (iii) it is desirable to examine whether such plasma miRNA change in patients undertaking various treatments. Nevertheless, it is worthy in the future to identify novel plasma miRNAs or non-coding RNAs using next-generation sequencing technologies.

In conclusion, elevation of miR-16, miR-21 and miR-451 in plasma of breast cancer patients has been reported in this study. Strikingly, we are the first to demonstrate that reduction of plasma miR-145 level occurred in breast cancer patients. The best combination of plasma markers miR-145 and miR-451 in our plasma quantitative assay makes a very promising and specific breast cancer screening test possible. Although the testing with these markers reach sensitivity and specificity >90% for breast cancer prediction, the positive predictive value was around 90% and the negative predictive value was around 92%, a combination of other potential markers using this plasma quantitation may further enhance the discriminating power of this test in the future.

## Supporting Information

Figure S1 **Box plot of RNU6B Ct values in the plasma from Normal control and breast cancer (BC) patients.** The lines inside the boxes denote the medians. The boxes mark the interval between the 25th and 75th percentiles. The whiskers denote the interval between the 10th and 90th percentiles. Filled circles indicate data points outside the 10th and 90th percentiles.Statistically significant differences were determined using Mann-Whitney test. NS, non-significant.
(TIF)

Figure S2 **Box plots of plasma levels of miR-16, miR-451 and miR-21 in female (F, n = 60) and male (M, n = 40) healthy subjects.** Expression levels of the miRNAs (Log10 scale at Y-axis) are normalized to RNU6B. Statistically significant differences were determined using Mann-Whitney test. NS, non-significant.
(TIF)

Figure S3 **Box plot of combined plasma miR-145 and miR-451 levels across stage of breast cancer patients.** Expression levels of the miRNAs (Log10 scale at Y-axis) are calculated by the equation 2 -deltaCt. deltaCt was calculated by subtracting the Ct values of miR-145 from the Ct values of the miR-451. The lines inside the boxes denote the medians. The boxes mark the interval between the 25th and 75th percentiles. The whiskers denote the interval between the 10th and 90th percentiles. Filled circles indicate data points outside the 10th and 90th percentiles. Statistically significant differences were determined using Mann-Whitney tests.
(TIF)

Figure S4 (A) ROC analysis using combined plasma miR-145 and miR-451 for discriminating DCIS cases from normal subjects. (B) Box plots of combined plasma levels of miR-145 and miR-451 in various DCIS graded patients. Expression levels of the miRNAs (Log10 scale at Y-axis) are calculated by the equation 2 -deltaCt. deltaCt was calculated by subtracting the Ct values of miR-145 from the Ct values of the miR-451.Statistically significant differences were determined using Mann-Whitney test.
(TIF)

Figure S5 ROC analysis using combined plasma miR-145 and miR-451 for discriminating BC cases from normal subjects in the blind validation.
(TIF)

Figure S6 **Box plot of combined serum miR-145 and miR-451 levels from Normal control and breast cancer (BC) patients.** Expression levels of the miRNAs (Log10 scale at Y-axis) are calculated by the equation 2 -deltaCt. DeltaCt was calculated by subtracting the Ct values of miR-145 from the Ct values of the miR-451. The lines inside the boxes denote the medians. The boxes mark the interval between the 25th and 75th percentiles. The whiskers denote the interval between the 10th and 90th percentiles. Filled circles indicate data points outside the 10th and 90th percentiles. Statistically significant differences were determined using Mann-Whitney test.
(TIF)

Table S1 MiRNA-specific primer sequences for qRT-PCR.
(DOC)

Table S2 The list of upregulated miRNAs (change >2 fold as a cutoff level) identified using real-time PCR based miRNA profiling arrays in plasma and biopsy samples of 5 breast cancer patients compared to 5 healthy controls. Highlighted in red are the markers up-regulated in both plasma and breast cancer tumors.
(DOC)

Table S3 The list of downregulated miRNAs (change >2 fold as a cutoff level) identified using real-time PCR based miRNA profiling arrays in plasma and biopsy samples of 5 breast cancer patients compared to 5 healthy controls. Highlighted in red are the markers down-regulated in both plasma and breast cancer tumors.
(DOC)
