# Supplementary figures and images for: Circulating microRNAs as Specific Biomarkers for Breast Cancer Detection
Source: PLoS One. 2013 Jan 3;8(1):e53141. doi: 10.1371/journal.pone.0053141 (PMC3536802; doi:10.1371/journal.pone.0053141)

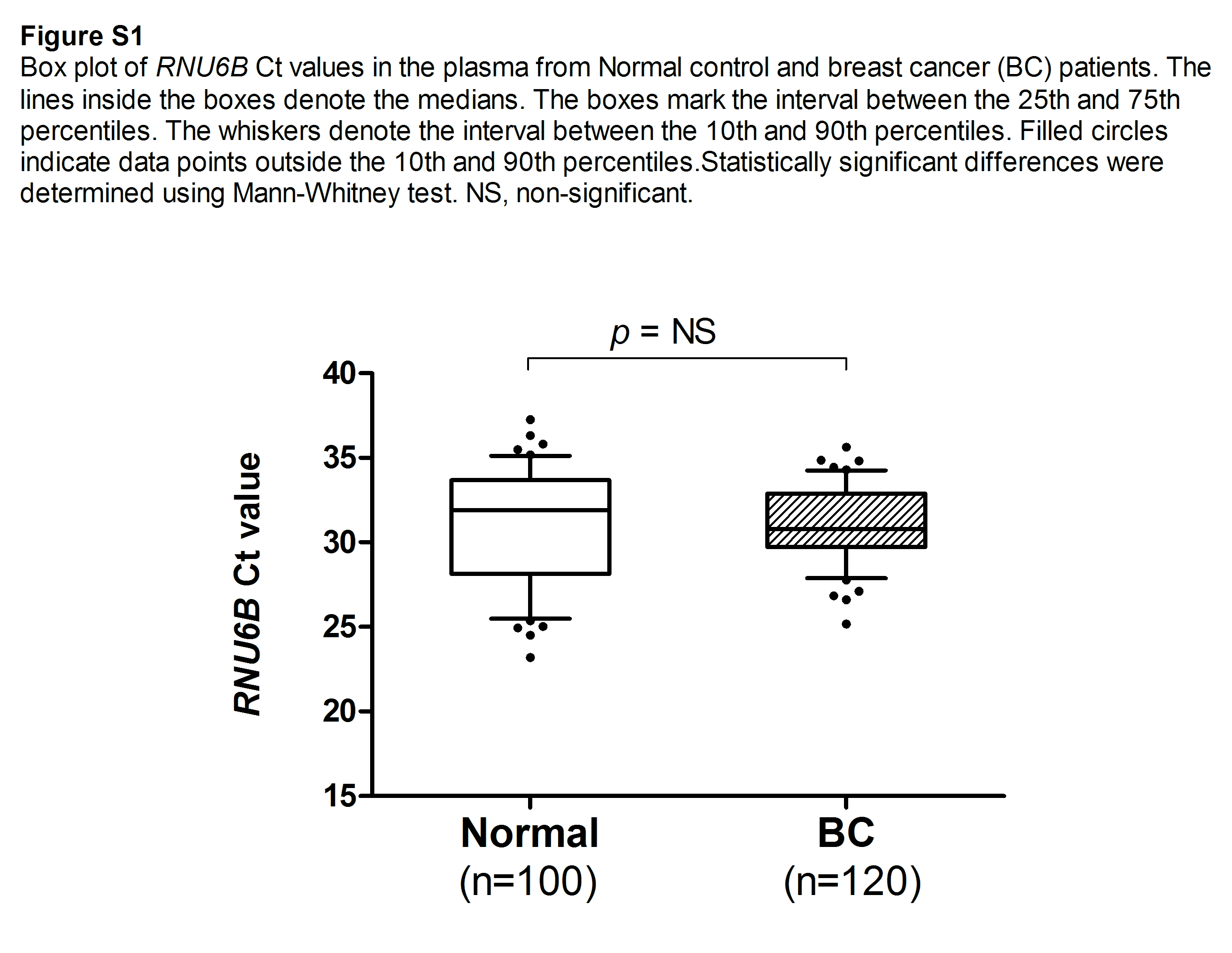

Supplement: Figure S1 — Box plot of RNU6B Ct values in the plasma from Normal control and breast cancer (BC) patients. The lines inside the boxes denote the medians. The boxes mark the interval between the 25th and 75th percentiles. The whiskers denote the interval between the 10th and 90th percentiles. Filled circles indicate data points outside the 10th and 90th percentiles.Statistically significant differences were determined using Mann-Whitney test. NS, non-significant. (TIF) [file pone.0053141.s001.tif]

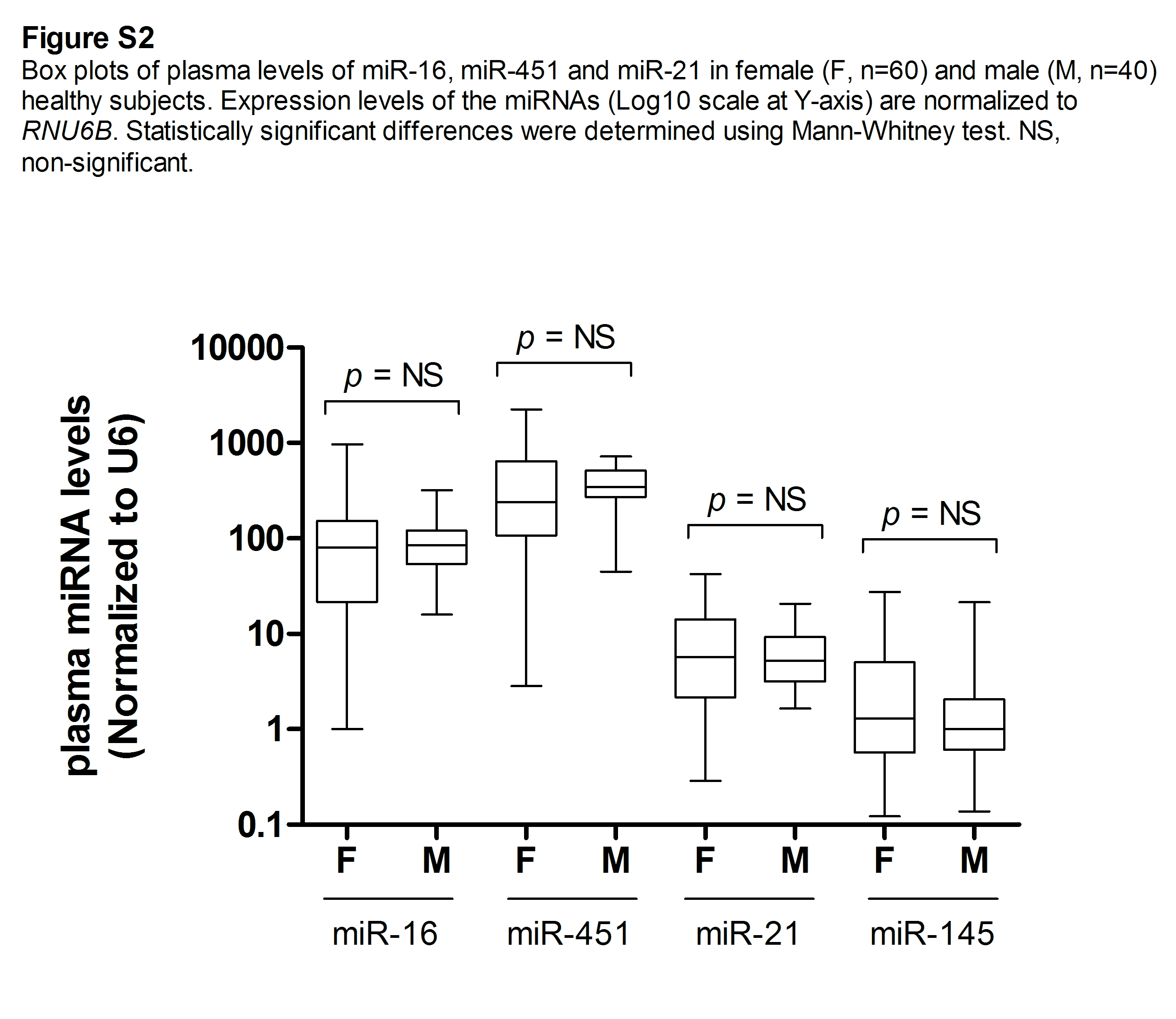

Supplement: Figure S2 — Box plots of plasma levels of miR-16, miR-451 and miR-21 in female (F, n = 60) and male (M, n = 40) healthy subjects. Expression levels of the miRNAs (Log10 scale at Y-axis) are normalized to RNU6B. Statistically significant differences were determined using Mann-Whitney test. NS, non-significant. (TIF) [file pone.0053141.s002.tif]

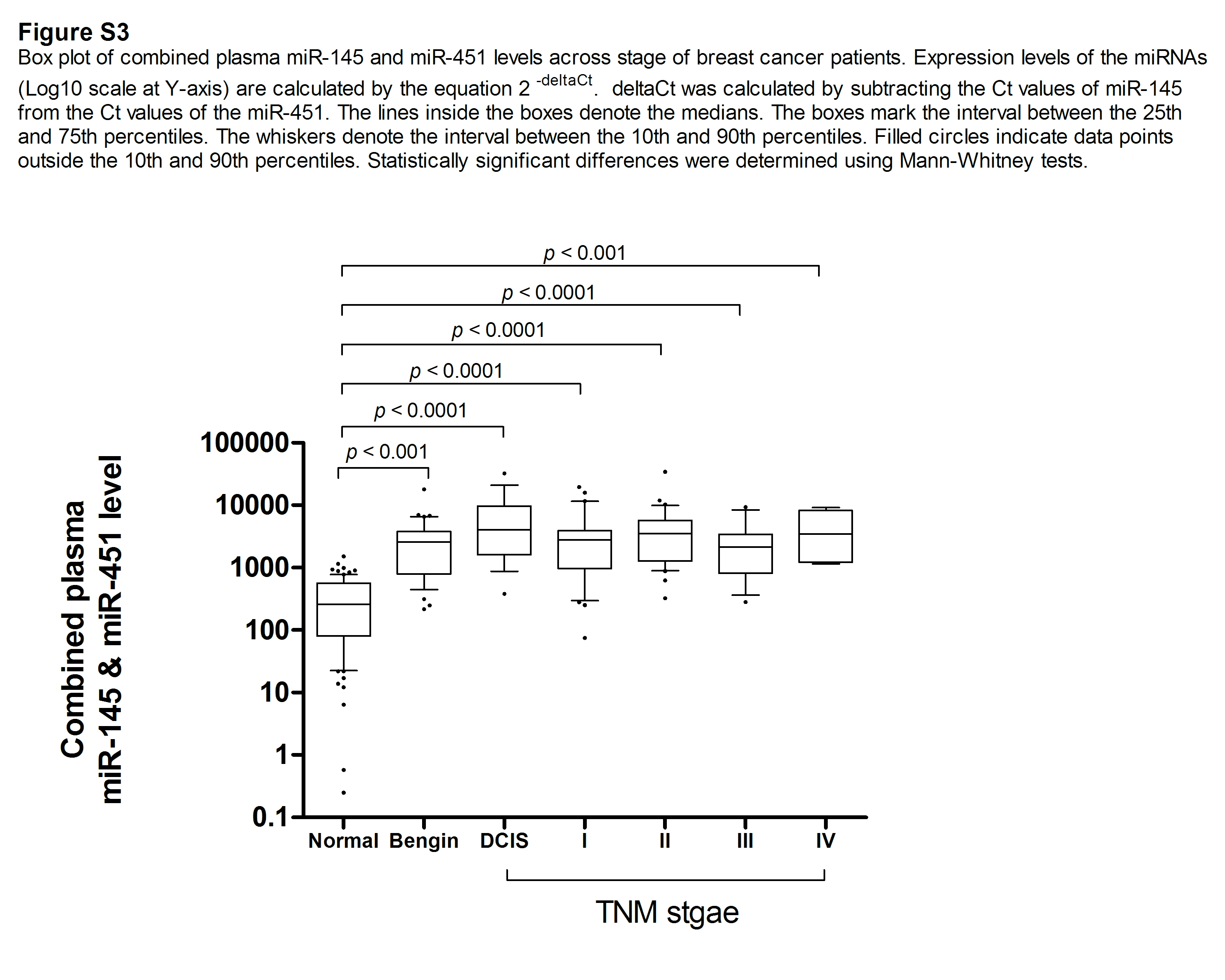

Supplement: Figure S3 — Box plot of combined plasma miR-145 and miR-451 levels across stage of breast cancer patients. Expression levels of the miRNAs (Log10 scale at Y-axis) are calculated by the equation 2 -deltaCt. deltaCt was calculated by subtracting the Ct values of miR-145 from the Ct values of the miR-451. The lines inside the boxes denote the medians. The boxes mark the interval between the 25th and 75th percentiles. The whiskers denote the interval between the 10th and 90th percentiles. Filled circles indicate data points outside the 10th and 90th percentiles. Statistically significant differences were determined using Mann-Whitney tests. (TIF) [file pone.0053141.s003.tif]

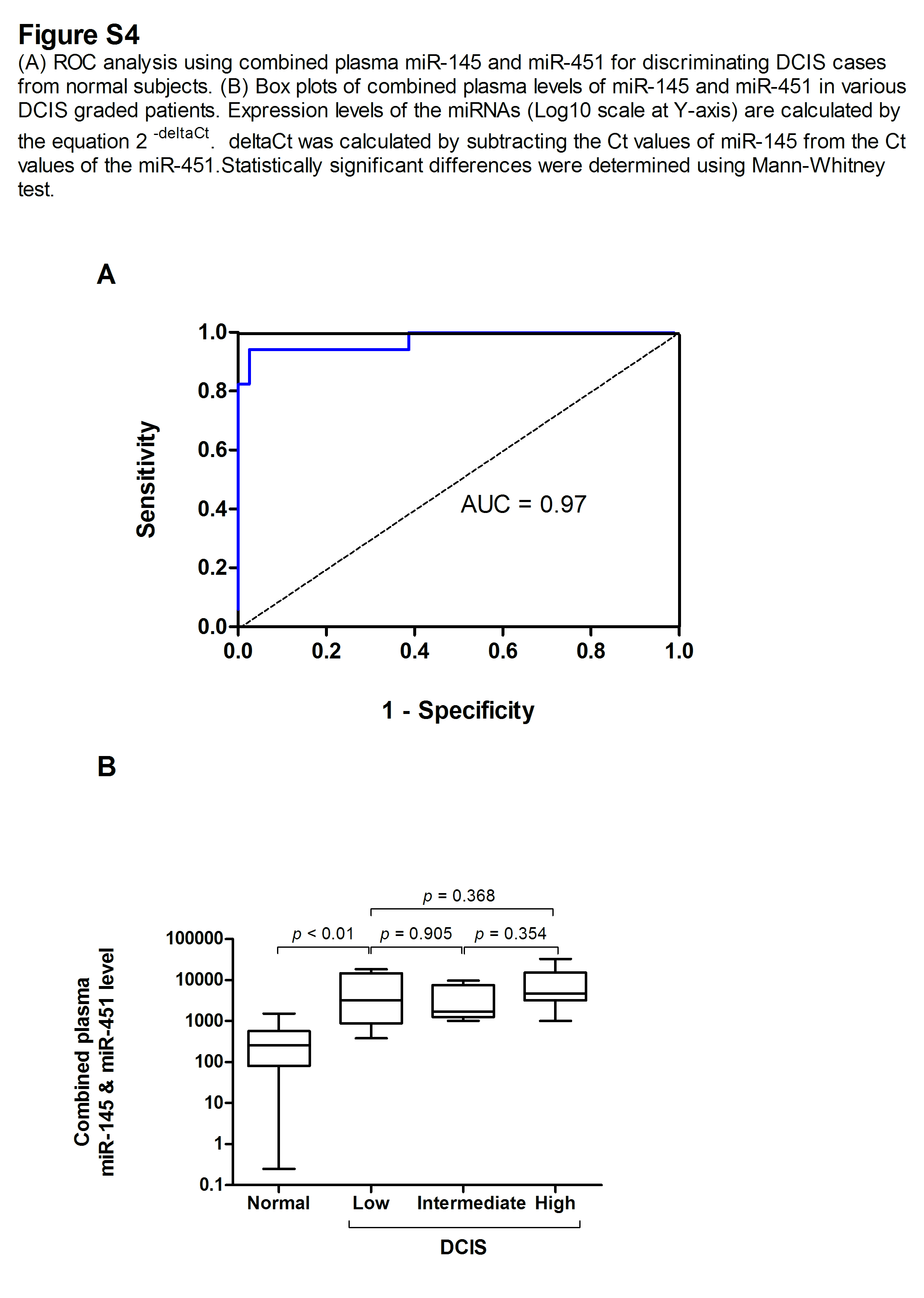

Supplement: Figure S4 — (A) ROC analysis using combined plasma miR-145 and miR-451 for discriminating DCIS cases from normal subjects. (B) Box plots of combined plasma levels of miR-145 and miR-451 in various DCIS graded patients. Expression levels of the miRNAs (Log10 scale at Y-axis) are calculated by the equation 2 -deltaCt. deltaCt was calculated by subtracting the Ct values of miR-145 from the Ct values of the miR-451.Statistically significant differences were determined using Mann-Whitney test. (TIF) [file pone.0053141.s004.tif]

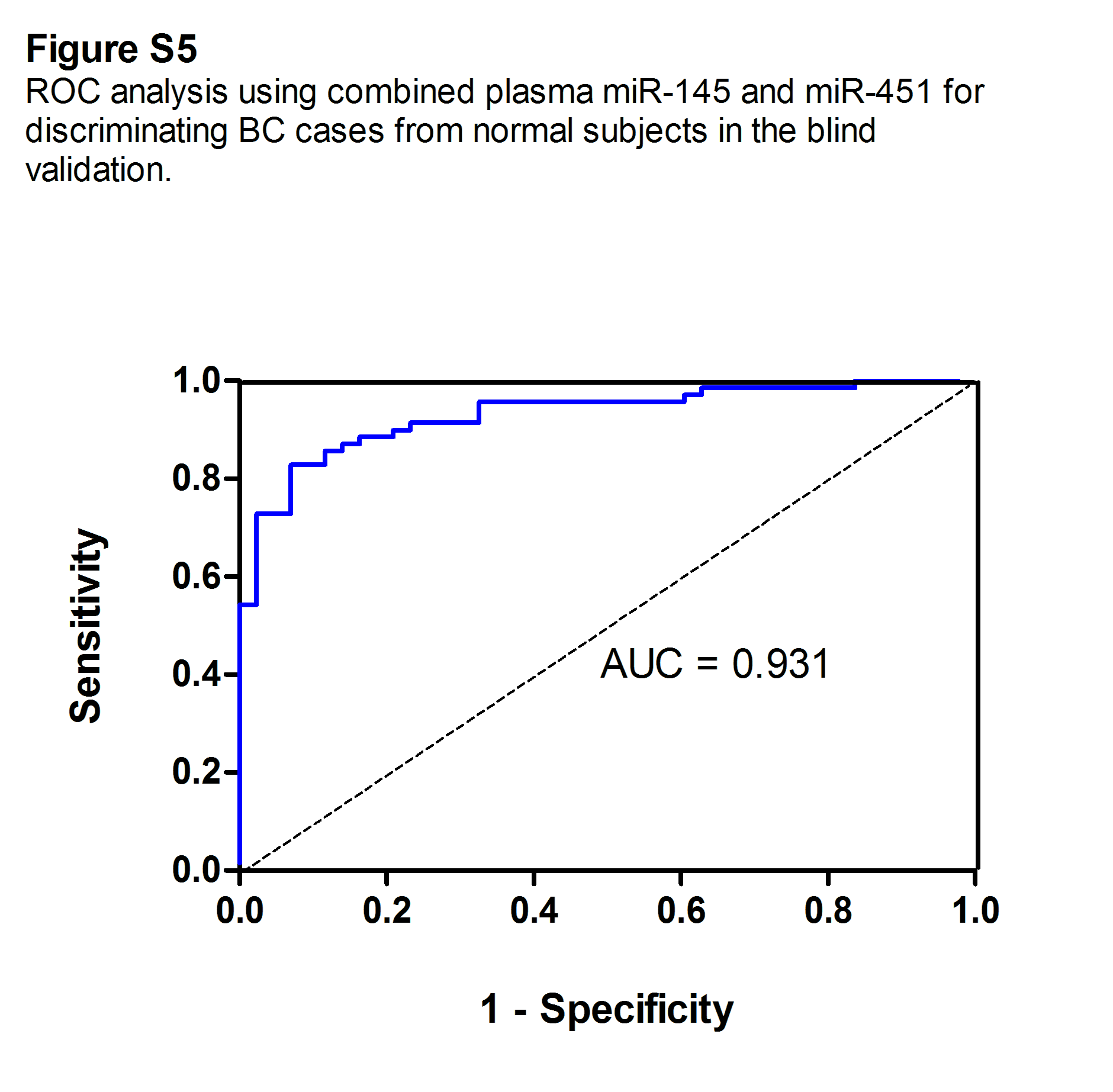

Supplement: Figure S5 — ROC analysis using combined plasma miR-145 and miR-451 for discriminating BC cases from normal subjects in the blind validation. (TIF) [file pone.0053141.s005.tif]

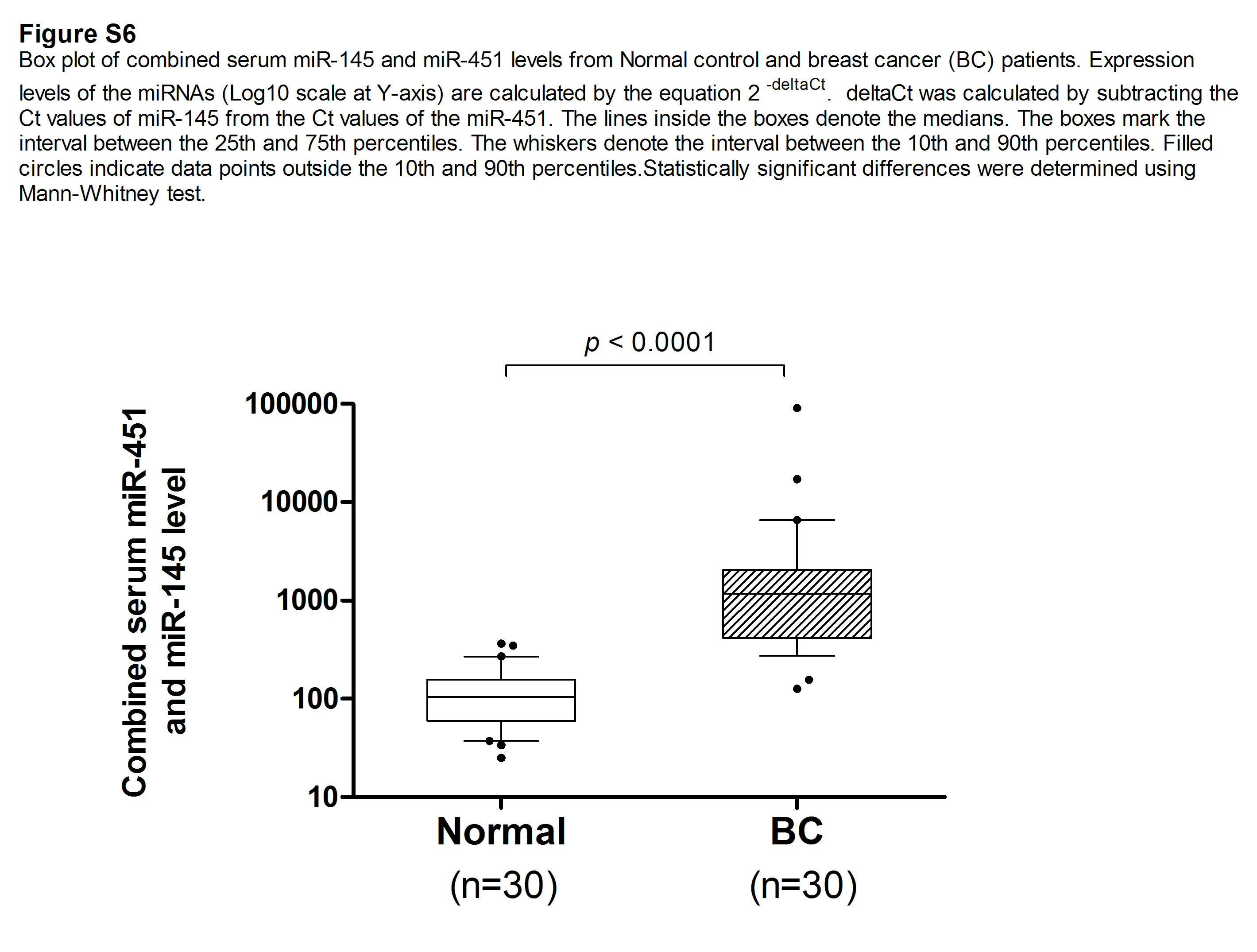

Supplement: Figure S6 — Box plot of combined serum miR-145 and miR-451 levels from Normal control and breast cancer (BC) patients. Expression levels of the miRNAs (Log10 scale at Y-axis) are calculated by the equation 2 -deltaCt. DeltaCt was calculated by subtracting the Ct values of miR-145 from the Ct values of the miR-451. The lines inside the boxes denote the medians. The boxes mark the interval between the 25th and 75th percentiles. The whiskers denote the interval between the 10th and 90th percentiles. Filled circles indicate data points outside the 10th and 90th percentiles. Statistically significant differences were determined using Mann-Whitney test. (TIF) [file pone.0053141.s006.tif]
