# Supplementary material for: Circulating microRNAs as Specific Biomarkers for Breast Cancer Detection
Source: PLoS One. 2013 Jan 3;8(1):e53141. doi: 10.1371/journal.pone.0053141 (PMC3536802; doi:10.1371/journal.pone.0053141)
Supplement: Table S1 — MiRNA-specific primer sequences for qRT-PCR. (DOC) [file pone.0053141.s007.doc]

**Table S1:** MiRNA-specific primer sequences for qRT-PCR

| **MicroRNAs** | **MirBase accession no.** | **Primer sequences (5' to 3')** |
| --- | --- | --- |
| miR-16 | [MIMAT0000069](http://www.mirbase.org/cgi-bin/mature.pl?mature_acc=MIMAT0000069) | TAGCAGCACGTAAATATTGGCG |
| miR-21 | [MIMAT0000076](http://www.mirbase.org/cgi-bin/mature.pl?mature_acc=MIMAT0000076) | TAGCTTATCAGACTGATGTTGA |
| miR-451 | [MIMAT0001631](http://www.mirbase.org/cgi-bin/mature.pl?mature_acc=MIMAT0001631) | AAACCGTTACCATTACTGAGTT |
| miR-145 | [MIMAT0000437](http://www.mirbase.org/cgi-bin/mature.pl?mature_acc=MIMAT0000437) | TCCAGTTTTCCCAGGAATCC |
