# Supplementary material for: Circulating microRNAs as Specific Biomarkers for Breast Cancer Detection
Source: PLoS One. 2013 Jan 3;8(1):e53141. doi: 10.1371/journal.pone.0053141 (PMC3536802; doi:10.1371/journal.pone.0053141)
Supplement: Table S2 — The list of upregulated miRNAs (change >2 fold as a cutoff level) identified using real-time PCR based miRNA profiling arrays in plasma and biopsy samples of 5 breast cancer patients compared to 5 healthy controls. Highlighted in red are the markers up-regulated in both plasma and breast cancer tumors. (DOC) [file pone.0053141.s008.doc]

**Table S2:** The list of upregulated miRNAs (change > 2 fold as a cutoff level) identified using real-time PCR based miRNA profiling arrays in plasma and biopsy samples of 5 breast cancer patients compared to 5 healthy controls. Highlighted in red are the markers up-regulated in both plasma and breast cancer tumors.

| **Tumor versus adjacent normal Tissue** | | | **Plasma from breast cancer versus normal** | |
| --- | --- | --- | --- | --- |
| miRNAs | Average fold change |  | miRNAs | Average fold change |
| hsa-miR-489 | 12.65 |  | hsa-let-7g | 6.28 |
| hsa-miR-15b | 12.55 |  | hsa-miR-10a | 5.88 |
| hsa-miR-671-3p | 12.35 |  | hsa-miR-154 | 5.69 |
| hsa-miR-429 | 12.15 |  | hsa-miR-148a | 4.88 |
| hsa-miR-375 | 12.14 |  | hsa-miR-150 | 4.86 |
| hsa-miR-200c | 12.07 |  | hsa-miR-16 | 4.78 |
| hsa-miR-487b | 11.67 |  | hsa-miR-186 | 4.48 |
| hsa-miR-212 | 11.46 |  | hsa-miR-193b | 4.28 |
| hsa-miR-200a | 11.24 |  | hsa-miR-25 | 3.97 |
| hsa-miR-323-3p | 11.08 |  | hsa-miR-27a | 3.63 |
| hsa-miR-210 | 10.24 |  | hsa-miR-328 | 3.57 |
| hsa-miR-200b | 10.11 |  | hsa-miR-451 | 3.11 |
| hsa-miR-9 | 10.01 |  | hsa-miR-485-3p | 2.91 |
| hsa-miR-339-5p | 9.99 |  | hsa-miR-486-3p | 2.86 |
| hsa-miR-362-5p | 9.98 |  | hsa-miR-574-3p | 2.51 |
| hsa-miR-342-3p | 9.96 |  | hsa-miR-21 | 2.34 |
| hsa-miR-660 | 9.52 |  | hsa-miR-200c | 2.18 |
| hsa-miR-191 | 9.09 |  | hsa-miR-210 | 2.18 |
| hsa-miR-202 | 8.85 |  | hsa-miR-191 | 2.09 |
| hsa-miR-103 | 8.60 |  |  |  |
| hsa-miR-150 | 8.46 |  |  |  |
| hsa-miR-376a | 8.20 |  |  |  |
| hsa-miR-495 | 8.03 |  |  |  |
| hsa-miR-451 | 7.63 |  |  |  |
| hsa-miR-185 | 6.77 |  |  |  |
| hsa-miR-532-5p | 6.20 |  |  |  |
| hsa-miR-95 | 6.07 |  |  |  |
| hsa-miR-339-3p | 5.38 |  |  |  |
| hsa-miR-181a | 4.92 |  |  |  |
| hsa-miR-340 | 4.58 |  |  |  |
| hsa-miR-590-5p | 4.35 |  |  |  |
| hsa-miR-146a | 4.23 |  |  |  |
| hsa-miR-148b | 3.98 |  |  |  |
| hsa-miR-134 | 3.91 |  |  |  |
| hsa-miR-203 | 3.59 |  |  |  |
| hsa-miR-21 | 3.48 |  |  |  |
| hsa-miR-149 | 3.37 |  |  |  |
| hsa-miR-374b | 3.10 |  |  |  |
| hsa-miR-146b-5p | 3.06 |  |  |  |
| hsa-miR-141 | 2.87 |  |  |  |
| hsa-miR-16 | 3.66 |  |  |  |
| hsa-miR-92a | 2.65 |  |  |  |
| hsa-miR-193a-3p | 2.46 |  |  |  |
| hsa-miR-625 | 2.10 |  |  |  |
| hsa-miR-27a | 2.04 |  |  |  |
