# Supplementary material for: Circulating microRNAs as Specific Biomarkers for Breast Cancer Detection
Source: PLoS One. 2013 Jan 3;8(1):e53141. doi: 10.1371/journal.pone.0053141 (PMC3536802; doi:10.1371/journal.pone.0053141)
Supplement: Table S3 — The list of downregulated miRNAs (change >2 fold as a cutoff level) identified using real-time PCR based miRNA profiling arrays in plasma and biopsy samples of 5 breast cancer patients compared to 5 healthy controls. Highlighted in red are the markers down-regulated in both plasma and breast cancer tumors. (DOC) [file pone.0053141.s009.doc]

**Table S3:** The list of downregulated miRNAs (change > 2 fold as a cutoff level) identified using real-time PCR based miRNA profiling arrays in plasma and biopsy samples of 5 breast cancer patients compared to 5 healthy controls. Highlighted in red are the markers down-regulated in both plasma and breast cancer tumors.

| **Tumor versus adjacent normal Tissue** | | | **Plasma from breast cancer versus normal** | |
| --- | --- | --- | --- | --- |
| **miRNAs** | **Average fold change** |  | **miRNAs** | **Average fold change** |
| hsa-miR-324-5p | -23.39 |  | hsa-miR-145 | -7.83 |
| hsa-miR-143 | -15.9 |  | hsa-miR-494 | -5.43 |
| hsa-miR-145 | -14.27 |  | hsa-miR-193a-5p | -4.65 |
| hsa-miR-452 | -10.58 |  | hsa-miR-874 | -4.07 |
| hsa-miR-224 | -6.93 |  | hsa-miR-126 | -2.73 |
| hsa-miR-31 | -6.75 |  | hsa-miR-320 | -2.55 |
| hsa-miR-744 | -3.92 |  | hsa-miR-106a | -2.2 |
| hsa-miR-139-5p | -2.95 |  | hsa-miR-324-3p | -2.08 |
| hsa-miR-708 | -2.94 |  |  |  |
| hsa-miR-335 | -2.75 |  |  |  |
